# Refractory Hypotension and Delayed Respiratory Failure in Amlodipine Toxicity: A Multimodal Management Approach

**DOI:** 10.7759/cureus.98430

**Published:** 2025-12-04

**Authors:** Maria Haq, Chima Iloanugo, Rabiu Momoh

**Affiliations:** 1 Critical Care, Medway Maritime Hospital, Gillingham, GBR; 2 Medicine, Maidstone and Tunbridge Wells NHS Trust, Kent, GBR

**Keywords:** amlodipine toxicity, calcium channel blockers, high-insulin euglycaemic therapy (hiet), intoxication, non-cardiogenic pulmonary edema, pharmaceutics, pulmonary edema, pulmonary vasodilation, toxicology

## Abstract

Pulmonary oedema is a rare but possible paradoxical complication following the withdrawal of high-dose insulin euglycemic therapy (HIET). In the context of calcium channel blocker (CCB) toxicity, other potential confounding factors that could account for delayed pulmonary oedema include inadvertent fluid overload from management of hypotension caused by CCB toxicity, delayed toxicodynamic effects on the pulmonary vasculature and the heart, or a previously unknown underlying cardiac dysfunction. We report a case involving a woman in her early 50s who developed a symptomatic pulmonary oedema following the discontinuation of HIET used in the management of refractory hypotension complicating a massive amlodipine intoxication. This unexpected deterioration occurred despite improved haemodynamics following HIET used in the management of amlodipine toxicity. We have reviewed this case in relation to the existing literature on delayed or paradoxical pulmonary oedema complicating CCB toxicity, to the benefit of clinical personnel managing future similar cases.

## Introduction

Calcium channel blocker (CCB) overdose can be life-threatening because it can severely affect the heart, blood vessels, and the body's energy balance. Both dihydropyridines (such as amlodipine and nifedipine) and non-dihydropyridines (including verapamil and diltiazem) exert their effects by inhibiting L-type calcium channels, thereby limiting calcium influx into cardiac and vascular smooth muscle cells [1]. Under therapeutic conditions, dihydropyridines predominantly influence vascular tone, whereas non-dihydropyridines primarily affect cardiac conduction and contractility. However, in overdose scenarios, this pharmacological selectivity diminishes, leading to bradycardia, reduced cardiac output, and profound vasodilation. These pathophysiological changes often culminate in distributive shock and metabolic acidosis, which may be resistant to conventional interventions such as intravenous fluids, calcium supplementation, vasopressors, and inotropic agents [2].

High-dose insulin euglycaemic therapy (HIET) has become a cornerstone in the management of severe CCB toxicity. CCBs inhibit calcium-dependent insulin release, causing hypoinsulinemia and reduced myocardial glucose uptake. Insulin therapy restores myocardial carbohydrate utilization, improving contractility and cardiac output. High doses (much higher than diabetic dosing) are used to overcome resistance and metabolic derangements in CCB toxicity. Euglycemia is maintained with dextrose infusion to prevent hypoglycemia. Although HIET is generally well tolerated, it necessitates large volumes of glucose infusion and carries risks of hypoglycaemia, hypokalaemia, and fluid overload [3].

Non-cardiogenic pulmonary oedema (NCPO) is a recognized but infrequent complication of amlodipine overdose. It is thought to result from excessive vasodilation, increased capillary permeability, and inflammatory responses [4]. Importantly, while HIET is widely used in treating severe CCB poisoning, there is limited literature describing the onset of pulmonary oedema following its discontinuation, making this case particularly noteworthy. Iatrogenic fluid overload, the underlying presence of an uncovered heart dysfunction, or a delayed temporal effect of the CCB toxicity would be other important differentials of delayed pulmonary edema in a case of a CCB overdose. 

## Case presentation

A 51-year-old female was brought to a hospital's emergency area by ambulance after intentionally ingesting 300 mg (30 x 10 mg tablets) of amlodipine. She had vomited three times while being assessed about three hours following this overdose. She was initially haemodynamically stable and required a litre per minute of supplementary oxygen to keep her pulse oximetry saturation above 94%. Her past medical history included hypertension, hyperlipidaemia, anaemia, hepatic steatosis, and Graves’ disease.

Approximately four hours post-ingestion, she became hypotensive, for which she received fluid boluses, intramuscular glucagon 1 mg stat, and 10 mL of IV 10% calcium gluconate to little effect. Recurrent persistent hypotension was noted with metabolic acidosis on blood gas analysis. A radial arterial cannulation was done to continuously invasively monitor her blood pressure readings, and a right internal jugular vein central venous access was done. Intravenous noradrenaline was commenced at 0.8 mcg/kg/min to maintain a mean arterial pressure >65 mmHg, and a sodium bicarbonate infusion was started. She was transferred to a critical care unit for more intensive monitoring and treatment.

Following discussion with a National Poisons Information Service, HIET was initiated the same evening, alongside 50% dextrose infusion and potassium replacements. The following morning, she was haemodynamically stable on weaning doses of noradrenaline infusion. On subsequent assessment, she appeared clinically dry with a mildly raised serum lactate of 2.8 mmol/L (see results of relevant blood studies done in Table 1), so she received intravenous human albumin followed by crystalloid boluses for volume resuscitation. HIET was discontinued later that day once blood pressure stabilised and no noradrenaline support was required.

**Table 1:** Relevant blood study results undertaken on this patient

Test study	Result	Reference range	Comment
Serum lactate	2.8 mmol/L	0.5-2.2 mmol/L	Mildly elevated
NT-Pro BNP	3872 pg/mL	<450 pg/mL	Markedly elevated

**Figure 1:**
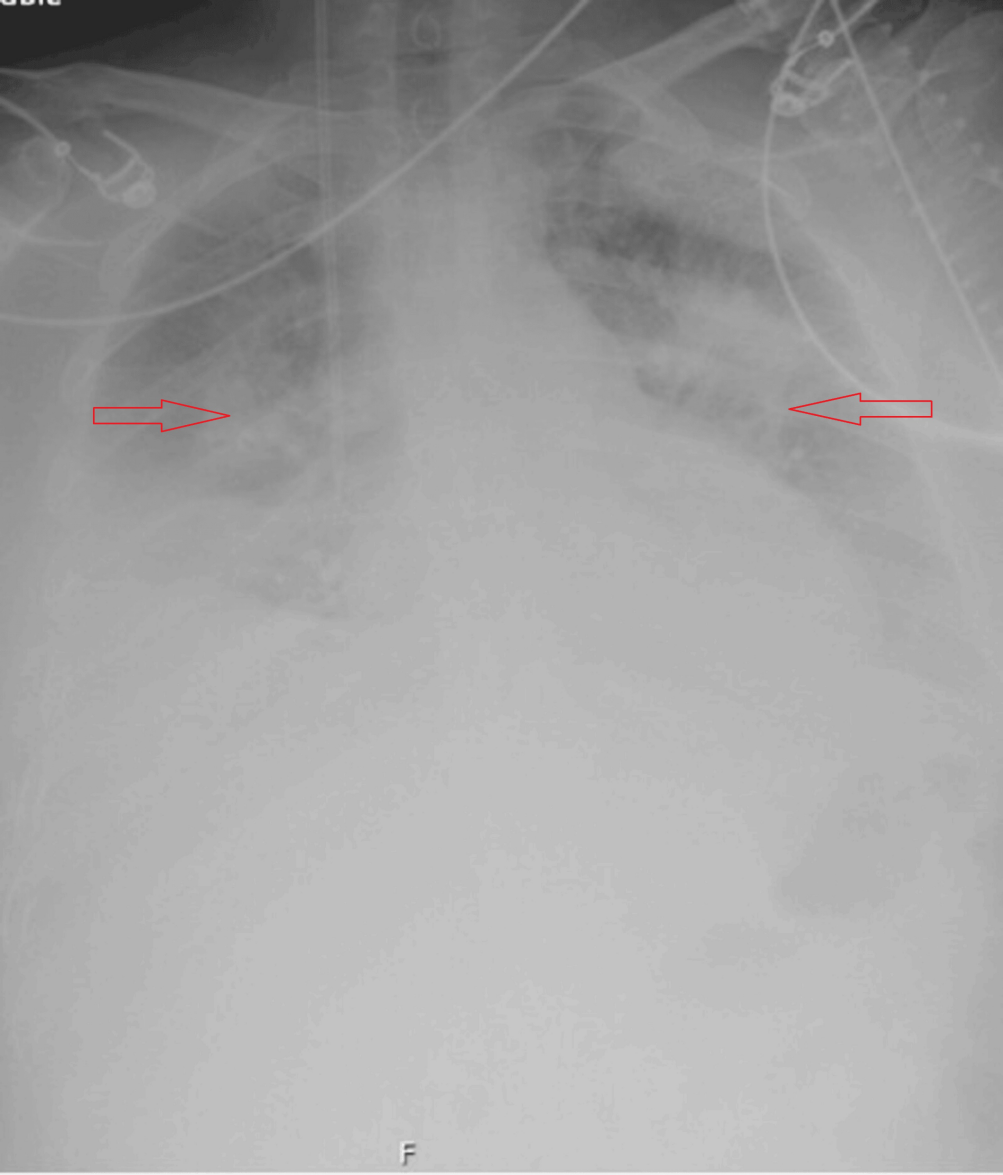
X-ray chest showing evidence of pulmonary congestion following high-dose insulin euglycemic therapy (HIET) in the treatment of calcium channel blocker (CCB) toxicity in the patient

**Figure 2:**
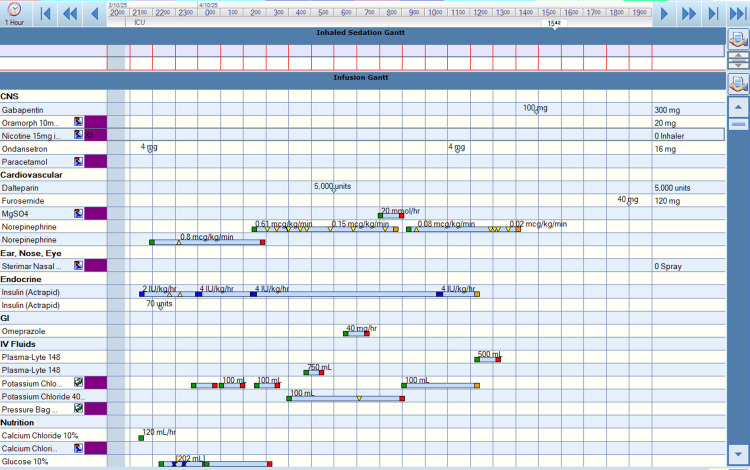
Pictorial trend of treatment offered to patient between day 0 and day 1 of gospital admission with amlodipine toxicity Image created by the authors using Metavision (iMDsoft, Tel Aviv, Israel)

After stopping HIET at about 19 hours post-intoxication, she developed new dyspnoea with hypoxaemic respiratory failure requiring high-flow nasal oxygen (FiO2 ~ 50%) within an hour (Figure 1). Chest radiography demonstrated diffuse opacification, suggesting pulmonary congestion (Figure 1). Bedside echocardiography showed preserved biventricular function, and she remained cardiovascularly unsupported. At the same time, she developed recurrent hypoglycaemia requiring ongoing 50% dextrose infusion and oral glucose administrations (see Figure 2). Her fluid balance on review at the time revealed a positive two litres value since admission to the hospital. Laboratory studies revealed that her serum creatinine, serum urea, and an estimated glomerular filtration rate were within normal limits. A continuous furosemide infusion was commenced, and respiratory support was escalated to continuous positive airway pressure (CPAP) via a tight-fitting face mask to maintain oxygen saturation. Dextrose infusion was gradually weaned as glucose stabilised.

Over the following two days, she continued to require high oxygen concentrations (FiO_2_ 55-80%) via a CPAP device. She remained cardiovascularly unsupported, and diuresis improved on furosemide infusion escalation up to 10 mg/hr. Pro-BNP (beta-natriuretic peptide) value check was 3,872 pg/mL (ref: <450 pg/mL), but formal echocardiography on day 2 confirmed preserved biventricular function. A rising C-reactive protein prompted empirical broad-spectrum antibiotics for possible hospital-acquired pneumonia.

CT pulmonary angiography (CTPA) on day 3 showed bilateral, moderately large pleural effusions, upper lobe ground-glass opacities, and bibasal consolidation, consistent with pulmonary oedema and inflammation. There was no evidence of acute or chronic pulmonary embolism. The patient remained on high-flow nasal oxygen therapy with intermittent CPAP (FiO2 ~65%). Diuresis continued.

By day 4, her oxygen requirement had decreased, with only mild basal crepitations on auscultation and a net negative fluid balance of -1.8L. The furosemide infusion was stopped, and by the afternoon she was saturating on room air and clinically stable for step-down to the respiratory ward. There, she was monitored. She was reviewed by a psychiatry unit and was discharged after five more days with outpatient follow-up plans. 

The timeline of her index hospital admission and interventions received is presented in Table 2. 

**Table 2:** Timeline of patient's admission and treatments received HIET: high-dose insulin euglycemic therapy, BP: blood pressure, CXR: chest X-ray, CRP: C-reactive protein, BNP: B-type natriuretic peptide, CTPA: computed tomography pulmonary angiography

Time from ingestion (overdose on amlodipine 300 mg)	Clinical events and findings	Interventions
~3 hours	Vomiting ×3, haemodynamically stable, mild hypoxaemia (SpO₂ <94% without O₂)	O₂ 1 L/min, cardiac monitoring
~4 hours	Hypotension, metabolic acidosis	Fluid boluses, IM glucagon 1 mg, IV calcium gluconate 10 mL (10%), arterial line, central line, IV noradrenaline (0.8 mcg/kg/min), sodium bicarbonate infusion
6 hours	Refractory hypotension despite noradrenaline infusion support	Transferred to ICU, HIET started with 50% dextrose + potassium replacement
18 hours	Haemodynamically stable, noradrenaline weaning	IV albumin + crystalloid boluses for resuscitation
~19 hours	HIET discontinued after BP stabilisation	—
Shortly after HIET stop	Dyspnoea, hypoxaemic respiratory failure, pulmonary congestion on CXR, recurrent hypoglycaemia	High-flow nasal O₂ (FiO₂ ~50%), CPAP, ongoing 50% dextrose + oral glucose, continuous furosemide infusion
Days 1–2	High O₂ requirement (FiO₂ 55–80%), diuresis with furosemide, CRP rise	Furosemide infusion escalated (up to 10 mg/hr), empirical broad-spectrum antibiotics
Day 2	BNP :3872 pg/mL, trans-thoracic echo: preserved biventricular function	Continued supportive care
Day 3	CTPA: bilateral pleural effusions, ground-glass opacities, bibasal consolidation (pulmonary oedema/inflammation), no pulmonary embolus found	High-flow O₂ + intermittent CPAP, ongoing diuresis
Day 4	Improved oxygenation, net negative fluid balance (-1.8 L), mild basal crepitations	Furosemide infusion stopped, saturating on room air, step-down to respiratory ward
Days 5–9	Stable on ward	Psychiatric review, monitoring
Day 9	Discharge	Outpatient follow-up arranged

## Discussion

We report a case of a 51-year-old woman who developed pulmonary oedema shortly after discontinuation of HIET following an intentional overdose of amlodipine. This presentation is notable for a possible delayed onset of NCPO, a complication not commonly reported after HIET cessation.

A retrospective analysis by Isbister et al. in the British Journal of Clinical Pharmacology highlighted the rising incidence of dihydropyridine CCB exposures, including amlodipine, due to their widespread therapeutic use [5]. Management of CCB toxicity typically involves supportive care - airway protection, intravenous fluids, calcium gluconate, atropine for bradycardia, vasopressors, and HIET. Adjuvant use of Intralipid is gaining traction in the management of CCB toxicity in the literature. In severe cases, mechanical ventilation and extracorporeal membrane oxygenation (ECMO) may be required [6].

HIET is recommended for patients with CCB toxicity who manifest with myocardial depression and acidosis. HIET involves insulin infusion at 0.5-1 U/kg/hr with glucose to maintain euglycemia. Insulin exerts positive inotropic effects without chronotropic action and promotes vasodilation via endothelial nitric oxide synthase (eNOS) activation through the PI3K pathway. While this improves cardiac output, it may also contribute to systemic hypotension and regional hypoperfusion [7]. Rietjens et al. demonstrated in a porcine model that combining HIET with vasopressors improves cerebral perfusion and reduces fluid overload [8].

NCPO is defined by alveolar fluid accumulation with a pulmonary artery wedge pressure ≤18 mmHg. Pulmonary artery wedge pressure, which would have been obtained via a pulmonary artery catheterization, was not studied in our patient. If studied, it would have been a useful assessment to delineate between cardiogenic and NCPO. Our patient had a raised pro-BNP value with a preserved biventricular function on trans-thoracic echocardiogram study, on further assessment for his pulmonary oedema occurrence. She had a normal renal function on laboratory blood assessments. The availability of serial pro-BNP studies and central venous pressure values would have added more information to the evaluation of this delayed pulmonary edema. Proposed mechanisms for this include precapillary vasodilation, increased hydrostatic pressure, and endothelial barrier disruption. The terminal half-life of amlodipine is approximately 30 -50 hours. Lindeman et al. reported a 47% incidence of NCPO in amlodipine poisoning cases. HIET may also exacerbate fluid overload due to large-volume dextrose infusions [9]. Schult et al. found that 60% of patients receiving HIET experienced volume overload [10].

In our case, HIET successfully stabilized haemodynamics during refractory shock. However, pulmonary oedema developed after HIET cessation, a phenomenon not previously described in the literature. This raises the possibility of rebound toxicity or delayed endothelial dysfunction. Some sources advocate gradual weaning of HIET to mitigate rebound effects, though insulin pharmacokinetics and haemodynamic impacts remain unpredictable [11]. Future research effort that would undertake advanced cardiovascular studies/monitoring to determine the cause of delayed pulmonary edema following CCB toxicity is suggested. 

The patient received HIET for 13 hours with concurrent noradrenaline, glucose, and potassium infusions. Hypoglycaemia persisted for two days post-HIET, requiring ongoing dextrose infusion. The relatively rapid onset of respiratory failure coincided with noradrenaline discontinuation, despite stable mean arterial pressure and preserved ventricular function. This suggests a multifactorial disruption of physiological balance.

Insulin-induced sodium retention and fluid accumulation may have contributed to subclinical pulmonary congestion. Once HIET was stopped, its vasodilatory and inotropic effects ceased, potentially increasing pulmonary capillary pressure. Given amlodipine’s long half-life, endothelial dysfunction may have persisted, facilitating capillary leak. The removal of noradrenaline may have further reduced systemic vascular resistance, promoting fluid redistribution into the lungs.

Emerging therapies such as hemo-adsorption have been proposed to accelerate toxin clearance and reduce HIET duration, thereby limiting fluid-related complications [12]. However, data on the timing and risk of pulmonary oedema post-HIET remain sparse. While hypotension rebound post-HIET discontinuation has been documented, delayed fluid overload following it is not yet fully characterized.

## Conclusions

The case report adds evidence to the multifactorial concept description of delayed pulmonary oedema complicating CCB overdose. It also highlights the complex interplay between CCB toxicity and insulin therapy. Further research is warranted to elucidate pathophysiology and optimize management strategies.
